# Safety evaluation of mutagenicity, genotoxicity, and cytotoxicity of *Lactobacillus* spp. isolates as probiotic candidates

**DOI:** 10.1002/jcla.24481

**Published:** 2022-05-17

**Authors:** Atieh Darbandi, Shiva Mirkalantari, Rezvan Golmoradi Zadeh, Maryam Esghaei, Malihe Talebi, Maryam Kakanj

**Affiliations:** ^1^ 440827 Microbial Biotechnology Research Centre Iran University of Medical Sciences Tehran Iran; ^2^ 440827 Department of Microbiology School of Medicine Iran University of Medical Sciences Tehran Iran; ^3^ 440827 Department of Virology Iran University of Medical Sciences Tehran Iran; ^4^ Food and Drug Laboratory Research Center Food and Drug Administration MOH&ME Tehran Iran

**Keywords:** probiotics, mutagenicity, genotoxicity, cytotoxicity, *Lactobacillus*

## Abstract

**Background:**

Probiotics promote a healthy balance of gut bacteria and have many beneficial effects on human digestive physiology. Although, few side effects of probiotics have been reported. This study aimed to assess the safety of five probiotic candidate *Lactobacillus* strains isolated from healthy individuals by examining mutagenicity, genotoxicity, and oral toxic effects.

**Methods:**

Five selected candidate probiotic (SCPs) strains were evaluated for genotoxicity (Ames test with *Salmonella typhimurium*), in vitro mammalian chromosome aberration test and an in vivo mouse micronucleus assay on peripheral blood of mice. To evaluate the oral dose toxicity, BALB/c mice models were treated repeatedly (2000, 1000, and 500 mg/kg body weight /day) for 28‐days.

**Results:**

The Ames test performed for two *S*. *typhimurium* strains TA 98 and TA100 (both in the absence and in the presence of S‐9 metabolic activation system) did not show an increase in reverse mutation because of exposure to the SCPs in any of the doses (5.0, 2.5, 1.25, 0.625, and 0.3125 mg/plate). There was no genotoxicity in the SCPs treatment in the vitro chromosome aberration assay with Chinese hamster ovary cells (CHO‐K1). In addition, none of the tested strains increased the frequency of micronucleated reticulocytes in reticulocytes, the SCPs with the studied doses caused no substantial variation in the experimental groups compared to the negative control group (*p* > 0.05). SCPs were not acutely toxic when administered to male and female BALB/c mice by single gavage at (2000, 1000, and 500 mg/kg b.w/day) with no mortality or clinical signs, change in body weight or macroscopic abnormalities were observed in this dose range.

**Conclusion:**

As a result, SCPs did not induce mutagenic potential in vitro with bacterial reverse mutation, clastogenicity, and in vivo tests in the ranges of concentrations evaluated in our study.

## Graphical abstract

This study showed that native Lactobacillus strains isolated from feces had no mutagenic or genotoxic effects at all doses tested using bacterial reverse mutation, chromosome aberration, micronucleus in mice, and oral toxicity assays. Investigating the mechanism of anti‐mutagenic properties may have important implications for future research on probiotic bacteria and their ability to reduce the risk of cancer. This study showed that these five native strains are safe at the highest dose level due to the absence of treatment‐related side effects. According to this study, native probiotic strains have a high potential for use in the food and pharmaceutical industries. Nevertheless, in‐depth studies are recommended in clinical trials on 90‐day oral toxicity.

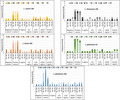

## 1 INTRODUCTION

Probiotic bacteria are microorganisms that have a wide range of health benefits for the host, including protection against direct colonization of the gastrointestinal tract (GI), maintaining the equilibrium of intestinal microflora, controlling digestion, and stimulating the immune system.^1^ Many lactic acid bacteria (LAB) strains are considered safe in most cases, given their long history of usage.^2^ Among all LAB, *Lactobacillus* is considered the largest genus generally recognized as safe (GRAS) and induces several health benefits on the host as a probiotic.^3^ In recent years, the use of live bacterial species such as *Lactobacillus* spp. due to safety benefits has received much attention. The GI is the location of too many bacterial species with critical functions in maintaining GI functionality, accounting for 70% of the human immune system functions.^4^ Moreover, in the European Union, 36 *Lactobacillus* strains are headed with Qualified Presumption of Safety (QPS).^5^ Several studies have shown that different strains of LAB have anti‐tumor and anti‐mutagenic properties, which are the chief characteristics of probiotics that could increase human lifespan.^6^

Nowadays, studies show that new or specific species of probiotics are constantly being identified. The efficacy of new strains must be carefully evaluated before incorporating them into products, and a case‐by‐case evaluation must be performed to determine whether they share the safety status of traditional organisms with food grade and whether probiotics have the potential to enter the industry.^7^, ^8^, ^9^

This is very important for humans because of the growing concern about the safety associated with probiotics whose extracts are being tested for their possible harmful effects on the host DNA.^10^, ^11^, ^12^ Presently, at least three actual tests are used universally to check the genotoxicity of different compounds. Bacterial gene mutations, chromosomal aberration assays in mammalian cells, and micronucleus tests in rodents as the fast and trustworthy tests are usually employed for genotoxicity investigation.^13^

Studies have shown that in each community, specific species could show better and more beneficial probiotic effects.^14^ Therefore, in this study, five *Lactobacillus* strains as a selective candidate probiotics (SCPs) from the previous study^15^ were assessed in terms of genetic toxicity, their potential mutagenic effects according to OECD guidelines (Organization for Economic Cooperation and Development) by performing laboratory evaluations and in vivo studies using mouse models to depict the safety of the strain in question.

## 2 MATERIAL AND METHODS

### 2.1 Ethics statement

The Ethics Committee approved all procedures and techniques used in this research of the Iran University of Medical Sciences (ethical code: IR.IUMS.FMD. REC1396.C464). All toxicity tests on these strains were performed in the microbiology laboratory based on protocols of OECD (ENV/MC/CHEM (98)17, 1997), FDA (21 CFR Part 58, 2014), and DOM (Department of Microbiology) in Iran.

All animal procedures and techniques were performed under ethical standards and approved by the Animal Care and Use Committee of Iran University of Medical Sciences (ethical code: IR.IUMS.FMD.REC1396.C464).

### 2.2 Bacterial strains and growth conditions

Five *Lactobacillus* strains (SCPs), including two strains of *Lactiplantibacillus plantarum* (*L*. *plantarum* 42) and (*L*. *plantrum* 165), one strain of *Levilactobacillus brevis* (*L*. *brevis* 205), *Limosilactobacillus reuteri* (*L*. *reuteri* 100), and *Lacticaseibacillus rhamnosus* (*L*. *rhamnosus*195) were isolated from stool were selected according to the results of our previous studies investigating the probiotic properties of these strains. These strains were isolated from 3 males and 2 females; their ages ranged from 1 to 36.

Inclusion criteria were; healthy humans without present or past underlying diseases, lack of any antibiotic therapies over 3 months prior to sampling, and no gastrointestinal (GI) disorders or consumed probiotics at the sampling time. Smokers, alcohol users, and people with underlying diseases were excluded from the study. Phenotypic methods identified the isolates as described previously^15^

SCPs were used as a lyophilized powder with a concentration of 4 × 10^11^ CFU/g. The isolates grew in deMan, Rogosa, and Sharpe medium (MRS; Sigma, Aldrich, UK) at 37°C for 18 h. After incubation, the broth was centrifuged at 4000 × *g* for 12 min at 4°C, the supernatants of SCPs were discarded, and the cell pellets were freeze‐dried and stored at −20°C until use.

### 2.3 Bacterial reverse mutation test

Ames test was performed following the protocols of the OECD guideline No. 471 (1997) and Maron and Ames (1983).^9^, ^10^ Ames test was employed to evaluate the potency of five *Lactobacillus* strains in inducing genetic mutation in *S*. *typhimurium* strains TA98 and TA100 (National Collection of Industrial Microorganisms, Iran).

*S*. *typhimurium* TA 98, TA100 and S‐9 (a metabolic activator of rat liver), were kept in a deep freezer at −80°C until utilized. For the preparation of metabolic activation, male rats (body weight~300g) were treated with 40 mg/kg sodium phenobarbitone (in 0.9% w/v saline) on day one and 60 mg/kg on days 2, 3, and 4. Then, five days later, animals were sacrificed and the livers were collected, homogenized in 0.18 M KCl. The homogenate was centrifuged at 12,000 *g* for 15 min. The supernatant was aliquoted (2 ml portions) and stored at −80°C. The S9 mix was prepared according to the recipe recommended by Maron and Ames, Mortelmans, and Zeiger.^3^, ^16^, ^17^ It consists of 10 mM of MgCl, 36 mM of KCl, 6 mM of glucose‐6‐phosphate, 4 mM of NADPH, and 3 mM of NADH, and 0.2 M sodium phosphate (pH 7.4) was added by 2 ml of S9.

Genotypes of *S*. *typhimurium* strains TA98 and TA100 were confirmed by examining histidine requirement, *rfa* mutation, *uvr*B mutation, and ampicillin resistance (Table 1). SCPs were used as lyophilized powders at the concentration of 4 × 10^11^ CFU/g and were dissolved in phosphate‐buffered saline (PBS) in five concentrations (5.0, 2.5, 1.25, 0.625, and 0.3125 mg/plate).

**TABLE 1:** Genotyping of the test bacterial strains

Strains	Histidine Requirement	*ΔuvrB* Mutation	*rfa* Mutation	Ampicillin Resistance
TA98	+	+	+	+
TA100	+	+	+	+

Briefly, about 100 μl of SCPs solution were mixed with 100 μL of an overnight culture of *S*. *typhimurium* strains TA98 or TA100 in either 0.5 ml of phosphate buffer (−S9 group) or 0.5 ml of S9 mix (+S9 group). The compound was blended with 3 ml of a molten top agar solution containing histidine/biotin agar (Sigma‐Aldrich), kept at 45 ± 5°C, and transferred to minimal‐glucose agar (MGA) plates. Then solidified agar plates were incubated at 37 ± 2°C in an incubator for 46 ± 2 h before counting the colonies. Positive and negative controls were mutagens included 4‐nitro‐o‐phenylenediamine (NPD), sodium azide (SA), benzo [a] pyrene (BP), and 2‐aminofluorene (2‐AF) (Sigma Chemical Co.), and phosphate‐buffered saline (PBS), respectively (Table 2). This test was performed in triplicate.

**TABLE 2:** Positive controls of Ames test

TA Strain	Without S9 activation	μg/plate	With S9 activation	μg/plate
TA98	4‐nitro‐o‐Phenylenediamine (NPD)	10.0	Benzo[*a*]pyrene (BP)	4.0
TA100	Sodium azide (SA)	0.4	2‐aminofluorene (2‐AF)	4.0

### 2.4 Chromosome aberration test

The principal media were bought from the Cell Bank of Pasteur Institute of Iran, including Chinese hamster ovary cells (CHO‐K1) (IBRC code: C10136) medium along with 10% fetal bovine serum (Biochrom), penicillin‐streptomycin solution, minimal‐glucose agar plates, master plates, soft agar, and nutrient broth. The CHO‐K1 cell doubling time for the 15th passage was determined to be 18 h, the cell culture for mycoplasma detection was negative.

First, for the cell viability test, the CHO‐K1 cell line was seeded into 96‐well plastic plates and treated with different SCPs concentrations with and without S9. Subsequently, in the treatment of 6 and 24 h, the media was removed and replaced with media containing 0.5 mg/ml of 3‐(4,5‐dimethylthiazol‐2‐yl)‐2,5‐diphenyltetrazolium bromide (MTT) solution to each well for another 1 h. Formazan crystals dissolved by Dimethyl sulfoxide and the absorbance of the tests were measured ELISA reader (Diego) at 590 nm.

Chromosome aberration test (CA) was employed according to two global protocols, including OECD (test No. 473, 1997)^11^ and FDA GLP (US Food and Drug Administration's Good Laboratory Practice) guidelines (2007).^18^ Aberrant chromosomes were detected in CHO‐K1 cells treated with SCPs at five concentrations (5.0, 2.5, 1.25, 0.625, and 0.3125 mg/ml) for 6 h either in the presence or absence of S9 and for 24 h without S9. Besides, two mutagenic substances, including 2‐μMmitomycin C (MC; Sigma‐Aldrich) at two concentrations of (0.1 and 0.05 μg/ml) and 80‐μMcyclophosphamide monohydrate (CPP; Sigma‐Aldrich) were used as the positive controls.

CHO‐K1 cell suspensions (4.0 × 10^6^ cells/ml) were seeded into 60‐mm plates in Ham's F12K medium supplemented with 10% fetal bovine serum (FBS) (Biochrom) and 1% penicillin/streptomycin (Sigma‐Aldrich) at a volume of 5 ml and then cultured in a CO_2_ incubator at 37°C in the air with 5% CO_2_.

In brief, 100 μl of a 10 (μg/ml) colcemid solution (Karyo MAX Colcemid Solution, Gibco, Life Technologies) was added into the culture and incubated for 3 h. Cells were harvested by trypsinization and centrifugation (4000 RPM for 10 min), swollen by freshly prepared potassium chloride at 0.075 M, and fixed with ice‐cold freshly (3:1 solution of methanol and acetic acid). The three samples were allowed to be air‐dried and stained with 1% Giemsa solution for 10 min before microscopic observation.

Finally, using Olympus DP‐70 microscope (Olympus) 100 chromosomes in metaphase for each specimen, with a total of 200 cells/concentration (two specimens) at magnification of 100× were selected for more evaluation. The chromosomal aberrations were analyzed according to structural and numerical classification. The criteria for number and structure changes of chromosomes were interpreted according to (ISCN 2020: An International System for Human Cytogenomic Nomenclature (2020).^19^

### 2.5 Micronucleus assay in mice

This assay was performed in accordance with the OECD guideline No. 474 for the testing of chemicals.^12^ In this experiment, 125 male BALB/c mice (aged 6–7 weeks, weighting 28 ± 3 g) were maintained in groups of five per cage with aspen chips bedding (Nepco, U.S.A.) under controlled conditions with 12 h light at 22 ± 3°C and 40%–70% relative humidity. In this experiment, 25 mice were used to test each SCPs. All procedures were approved by the Animal Care and Use Committee of Iran University of Medical Sciences.

After one week of acclimatization to the environment, five mice were randomly assigned to each group and daily treated with three different concentrations of SCPs via oral gavage, including low dose (500 mg/kg b.w./day), middle dose (1000 mg/kg b.w./day), and high dose (2000 mg/kg b.w./day). Positive control was administered by mitomycin C (Sigma‐Aldrich) at a dose of 1 mg/kg via intraperitoneal injection. Reverse osmosis (RO) water was used as a negative control, and injected 15 ml/kg of the SCPs in all three doses by gavage to mice.

In this test, mice were anesthetized with isoflurane, and peripheral blood was sampled from the orbital sinus 48 and 72 h after receiving the first dose, and positive control was sampled only 48 h after the first dose.

Additionally, 36 h after the last day of administration, 3–4 μl of peripheral blood was collected from the mouse tail, placed on a slide to dry in the air, and fixed with methanol, then were incubated at room temperature for 1–2 h before examination with a light microscope. The ratio of reticulocytes (RETs) to micronucleated reticulocytes (MN) was analyzed in 1000 reticulocytes with Giemsa staining.^20^

### 2.6 Oral toxicity assay

A 28‐day oral toxicity study was performed on BALB/c mice in compliance with test No. 407.^21^ In the developmental toxicity survey, 20 male and 20 female BALB/c mice (aged 6–7 weeks, weighting 28 ± 3 g) were randomly assigned into groups of four per cage under controlled conditions with a 12‐h light/dark cycle at 23°C and 65% relative humidity. Animals experienced a 28‐day subacute toxicity survey and were treated with three different concentrations mixture of SCPs. Via daily oral gavage, including low dose (500 mg/kg b.w./day), middle dose (1000 mg/kg b.w./day), and high dose (2000 mg/kg b.w./day). Each mouse's body weight was measured two times a week and water and food consumption after 2 and 4 weeks. On day 29, mice were sacrificed. The mice's vital signs, including eyes, respiration, fur, movement, and weight, were monitored. The specific growth rate (SGR) was expressed as the average weekly weight gain (g). Specific growth mice = (*W* − *W*0)/*W*0 × 100 (%), *W*: mice weight on the defined feeding days; *W*0: mice weight on day 0.

### 2.7 Statistics

The collected data in this research were reported as mean ±SD. Data related to Ames test, body weight, and frequency of RETs and MN% were analyzed using SPSS software (IBM, NY, USA) by employing one‐way ANOVA and Duncan's multiple range assays. The significance of the differences between the groups was evaluated by *p *< 0.05.

## 3 RESULTS

### 3.1 Bacterial reverse mutation test

Genotypes of *S*. *typhimurium* strains TA98 and TA100 were confirmed at first step by examining histidine requirement, *rfa* mutation *uvrB* mutation, and ampicillin resistance genotype (Table 1). Strains TA98 and TA100 had all the characteristics consistent with the former documents.^22^ Bacterial reverse mutation assay was conducted to assess the genotoxicity of different doses of SCPs against two mutant *S*. *typhimurium* strains. After induction, an awaited increase was observed in revertant colonies in all positive control groups.

Compared to the negative control, after exposure of bacterial strains to SCPs with five concentrations (5.0, 2.5, 1.25, 0.625, and 0.3125 mg/plate), no significant (*p*<0.05) increase was observed in the number of revertant colonies neither in the presence (0.5 mL of S9 mix) nor in the absence of S9 (Table 3). Therefore, these five strains of SCPs were considered to have no mutagenic activity in histidine auxotrophy of *S*. *typhimurium* strains TA98 and TA100. All data were calculated as mean ±SD in three independent experiments (Table 3).

**TABLE 3:** Ames test results of SCPs using *S*. *typhimurium* strains TA98 and TA100

*Lactobacillus* (mg/plate)	*Lactobacillus plantrum 42*		*Lactobacillus rhamnosu 195*		*Lactobacillus brevis 205*		*Lactobacillus plantrum 165*		*Lactobacillus reuteri 100*	
	TA98	TA100	TA98	TA100	TA98	TA100	TA98	TA100	TA98	TA100
With S9	26 ± 6.3	120 ± 1.2	43 ± 2.8	151 ± 2.5	28 ± 2.5	205 ± 1.2	32 ± 2.1	201 ± 3.1	23 ± 5.2	178 ± 2.2
5.0	33 ± 4.0	128 ± 5.6	35 ± 2.3	140 ± 4.4	30 ± 3.3	198 ± 1.2	31 ± 2.2	205 ± 5.2	25 ± 1.2	180 ± 3.3
2.5	31 ± 2.0	180 ± 5.6	45 ± 1.5	141 ± *2*.*8*	27 ± 3.2	208 ± 5.2	38 ± 2.7	178 ± 5.3	22 ± 3.3	153 ± 2.3
1.25	24 ± 5.2	203 ± 4.5	38 ± 5.2	135 ± 5.5	33 ± 1.2	159 ± 3.2	39 ± 6.2	132 ± 1.2	25 ± 2.2	220 ± 1.2
0.625	25 ± 2.2	125 ± 3.3	28 ± 6.3	128 ± 1.1	33 ± 2.8	187 ± 2.5	25 ± 3.1	125 ± 2.3	31 ± 2.5	194 ± 5.2
0.312	35 ± 2.6	202 ± 4.6	30 ± 3.3	144 ± 6.1	25 ± 3.3	169 ± 5.2	22 ± 8.6	179 ± 4.3	33 ± 4.5	180 ± 1.2
Negative control^b^	32 ± 3.3	202 ± 1.2	28 ± 5.2	143 ± 2.3	27 ± 2.2	210 ± 6.3	22 ± 4.2	145 ± 5.6	25 ± 7.5	176 ± 2.2
Positive control^c^	0.41^a^ ± 580	07^a^ ± 880	0.41^a^ ± 647	1100 ± 0.41^a^	0.07^a^ ± 968	0.41^a^ ± 478	0.07^a^ ± 1020	7.7^a^ ± 309	0.27^a^ ± 998	0.87^a^ ± 687
Without S9										
5.0	23 ± 5.8	168 ± 2.3	59 ± 4.3	145 ± 3.3	42 ± 3.9	132 ± 2.3	48 ± 3.6	190 ± 2.2	29 ± 1.2	150 ± 1.2
2.5	29 ± 4.3	144 ± 3.2	49 ± 3.3	154 ± 4.3	44 ± 2.2	122 ± 2.2	50 ± 2.2	200 ± 2.3	33 ± 3.3	132 ± 4.1
1.25	31 ± 4.3	120 ± 3.3	56 ± 2.3	140 ± 4.4	29 ± 3.2	138 ± 2.2	65 ± 9.3	200 ± 1.2	49 ± 2.1	144 ± 8.2
0.625	21 ± 5.3	168 ± 6.3	55 ± 5.5	154 ± 3.3	33 ± 1.2	132 ± 5.6	54 ± 2.3	190 ± 2.3	31 ± 2.3	156 ± 1.2
0.312	22 ± 5.2	123 ± 3.2	48 ± 1.2	130 ± 7.1	29 ± 2.3	122 ± 3.3	48 ± 4.3	160 ± 2.3	30 ± 5.2	146 ± 5.3
Negative control^b^	28 ± 1.2	151 ± 5.5	49 ± 2.3	147 ± 8.3	33 ± 4.2	132 ± 2.3	54 ± 8.5	190 ± 2.2	28 ± 6.3	156 ± 2.3
Positive control^d^	987 ± 1.2^a^	445 ± 3.3^a^	655 ± 2.3^a^	930 ± 2.2^a^	1003 ± 3.3^a^	687 ± 1.2^a^	965 ± 6.6^a^	404 ± 7.5^a^	887 ± 5.3^a^	987 ± 3.3^a^

^a^ A significant difference compared to the negative control (<0.05).

^b^ Sterile water was used as a negative control.

^c^ Positive controls with S9 for TA98: Benzo[*a*]pyrene, 4.0 μg/plate; and for TA100: 2‐aminofluorene (2‐AF), 4.0 μg/plate.

^d^ Positive controls without S9 for TA98: 4‐nitro‐o‐phenylenediamine (NPD), 10.0 μg/plate; and for TA100: Sodium azide (SA), 0.4 μg/plate.

### 3.2 Chromosome aberration test

The primary toxicity experiment of SCPs with five concentrations (5.0, 2.5, 1.25, 0.625, and 0.3125 mg/plate) on cell culture under a 24 h continuous exposure protocol without S9 mix, and under 6 and 24 h exposure, both in the presence and in the absence of S9 mix, demonstrated that none of the *Lactobacillus* strains induced‐cytotoxicity. The survival ratio (relative to control) in all groups ranged from 61 to 100%, with no sign of a dose‐response relationship.

For the 6 h (absence and presence of S9 mixture), all SCPs in every group did not cause a significant increase in the frequency of aberrant cells or the number of polyploid cells. The results of metaphase analysis and the effects of *L*. *reuteri* 100 on cell survival in Tables 4 and 5 and other strains (*L*. *plantarum* 42, *L*. *rhamnosus* 195, *L*. *brevis* 205, *L*. *plantarum* 165) are presented in the supplementary table (S1–S4). The results of positive controls and negative controls confirmed the results of tests (Figure S1).

**TABLE 4:** The number of structural and numerical chromosome aberrations induced in CHO‐K1 cells with *Lactobacillus reuteri* 100 exposures for 6 h period in the absence of metabolic activation

*L*. *reuteri* 100 (mg/ml)	Number of cells with structural chromosome aberrations (% in brackets)							Number of cells with numerical aberrations (%)		Survival ratio (%)
	Observed	Chromatid Breaks	Chromatid Exchanges	Chromosome Breaks	Chromosome exchanges	Gaps or Others	Total	Polyploids	Endoreduplicated Cells	
Negative control	200	0 (0.0)	0 (0.0)	2 (1.0)	0 (0.0)	0 (0.0)	2 (1.0)	1 (0.5)	0 (0.0)	100
0.3125	200	0 (0.0)	0 (0.0)	0 (0.0)	0 (0.0)	1 (0.5)	1 (0.5)	1 (0.5)	0 (0.0)	88
0.625	200	0 (0.0)	0 (0.0)	0 (0.0)	0 (0.0)	0 (0.0)	0 (0.0)	0 (0.0)	0 (0.0)	72
1.25	200	0 (0.0)	0 (0.0)	0 (0.0)	0 (0.0)	2 (1.0)	2 (1.0)	2 (1.0)	0 (0.0)	71
2.5	200	0 (0.0)	0 (0.0)	0 (0.0)	0 (0.0)	0 (0.0)	0 (0.0)	0 (0.0)	0 (0.0)	85
5.0	200	0 (0.0)	0 (0.0)	0 (0.0)	0 (0.0)	0 (0.0)	0 (0.0)	0 (0.0)	0 (0.0)	70
Positive control (MMC at 0.1 μg /ml)	200	1 (0.5)	18 (9.0)	26 (13)	40 (20.0)	0 (0.0)	85 (42.5)	1 (0.5)	0 (0.0)	68

Abbreviation: MMC, mitomycin C.

**TABLE 5:** The number of structural and numerical chromosome aberrations induced in CHO‐K1 cells with *L*. *reuteri* 100 exposures for 6 h period in the presence of metabolic activation.

*L*. *reuteri* 100 (mg/ml)	Number of cells with structural chromosome aberrations (% in brackets)							Number of cells with numerical aberrations (%)		Survival ratio (%)
	Observed	Chromatid breaks	Chromatid Exchanges	Chromosome Breaks	Chromosome Exchanges	Gaps or Others	Total	Polyploids	Endoreduplicated Cells	
Negative control	200	0 (0.0)	0 (0.0)	1 (0.5)	1 (0.5)	0 (0.0)	2 (1.0)	1 (0.5)	0 (0.0)	100
0.3125	200	0 (0.0)	0 (0.0)	0 (0.0)	0 (0.0)	0 (0.0)	0 (0.0)	1 (0.5)	0 (0.0)	90
0.625	200	0 (0.0)	0 (0.0)	0 (0.0)	0 (0.0)	0 (0.0)	0 (0.0)	0 (0.0)	0 (0.0)	85
1.25	200	0 (0.0)	0 (0.0)	0 (0.0)	0 (0.0)	2 (1.0)	2 (1.0)	3 (1.5)	0 (0.0)	85
2.5	200	0 (0.0)	0 (0.0)	0 (0.0)	0 (0.0)	1 (0.5)	1 (0.5)	1 (0.5)	0 (0.0)	77
5.0	200	0 (0.0)	0 (0.0)	0 (0.0)	0 (0.0)	1 (0.5)	1 (0.5)	0 (0.0)	0 (0.0)	83
Positive control (CPP at 80 μM)	200	0 (0.0)	85 (42.5)	36 (18)	0 (0.0)	2 (1.0)	123 (61.5)	1 (0.5)	2 (1.0)	76

Abbreviation: CPP, cyclophosphamide monohydrate

Furthermore, using 24 h exposure (absence S9 mixture), SCPs in every group caused no significant increase in the incidence of structural chromosome aberrations or in the number of polyploid cells. The results of metaphase analysis and the effects of *L*. *reuteri* 100 on cell survival in Table 6 and other strains (*L*. *plantarum* 42, *L*. *rhamnosus* 195, *L*. *brevis* 205, *L*. *plantarum* 165) are presented in the supplementary tables (Tables S1–S4). As in the 6 h test, the positive control produced a clear increase in structural chromosome aberrations. Besides, the in vitro CHO‐K1 chromosome aberration assay showed no clastogenic potential of *L*. *plantarum* 42, *L*. *rhamnosus* 195, *L*. *brevis* 205, *L*. *plantarum* 165 and *L*. *reuteri* 100 strains.

**TABLE 6:** The number of structural and numerical chromosome aberrations induced in CHO‐K1 cells with *Lactobacillus reuteri* 100 exposures for 24 h in the absence of metabolic activation

*L*. *reuteri* 100 (mg/ml)	Number of cells with structural chromosome aberrations (% in brackets)							Number of cells with numerical aberrations (%)		Survival ratio (%)
	Observed	Chromatid breaks	Chromatid Exchanges	Chromosome breaks	Chromosome Exchanges	Gaps or Others	Total	Polyploids	Endoreduplicated Cells	
Negative control	200	2 (1.0)	0 (0.0)	0 (0.0)	0 (0.0)	0 (0.0)	2 (1.0)	2 (1.0)	0 (0.0)	100
0.3125	200	0 (0.0)	0 (0.0)	0 (0.0)	0 (0.0)	0 (0.0)	0 (0.0)	1 (0.5)	0 (0.0)	76
0.625	200	0 (0.0)	0 (0.0)	0 (0.0)	0 (0.0)	0 (0.0)	0 (0.0)	2 (1.0)	0 (0.0)	88
1.25	200	0 (0.0)	0 (0.0)	0 (0.0)	0 (0.0)	2 (1.0)	2 (1.0)	0 (0.0)	0 (0.0)	78
2.5	200	0 (0.0)	0 (0.0)	0 (0.0)	0 (0.0)	2 (1.0)	2 (1.0)	1 (0.5)	0 (0.0)	85
5.0	200	0 (0.0)	0 (0.0)	0 (0.0)	0 (0.0)	0 (0.0)	0 (0.0)	0 (0.0)	0 (0.0)	88
Positive control (MMC at 0.05 μg /ml)	200	96 (48)	0 (0.0)	0 (0.0)	38 (19.0)	20 (10.0)	154 (77.0)	2 (1.0)	0 (0.0)	72

Abbreviation: MMC, mitomycin C.

### 3.3 Micronucleus assay in mice

There were no signs of toxicity after consuming low (500 mg/kg b.w./day), middle (1000 mg/kg b.w./day), and high (2000 mg/kg b.w./day) doses of SCPs for three consecutive days. No significant mortality, clinical symptoms, or abnormal bodyweight changes were observed during this trial period.

In the positive control group treated with mitomycin C, the frequency of RETs among total erythrocytes was 16 + 1.2 and 9 + 1.2 after 48 and 72 h, respectively, which were significantly lower than those in the negative control during the same period (Table 7). The frequency of RETs among total erythrocytes did not change in animals treated with different doses of SCPs, and no significant difference was observed compared to the negative control group (*p* < 0.05). On the other hand, the frequency of micronucleated RETs in the positive control treated with mitomycin C significantly increased after 48 h (18 + 1.3; *p* < 0.05) compared to the negative control and the experimental groups (Table 7). In addition, no significant increase was observed in the percentage of MN in RETs in animals treated with different doses of SCPs, and no significant difference was observed compared to the negative group (*p* > 0.05).

**TABLE 7:** Changes of micronucleus and reticulocyte counts in peripheral blood of mice treated with SCPs

*Lactobacillus* spp. Dose (mg/ml)	Time (h)	*Lactobacillus reuteri* 100		*Lactobacillus plantarum* 42		*Lactobacillus plantarum* 165		*Lactobacillus brevis* 205		*Lactobacillus rhamnosus*195	
		RET/1000 RBC%	MN/2000 RET%	RET/1000 RBC%	MN/2000 RET%	RET/1000 RBC%	MN/2000 RET%	RET/1000 RBC%	MN/2000 RET%	RET/1000 RBC%	MN/2000 RET%
Low (500 mg/kg b.w./day)	48	35 ± 4.4 (30.6–39.4)	1.2 ± 0.9 (0.3–2.1)	29 ± 3.1 (26.0–32.1)	1.2 ± 0.9 (0.3–2.1)	30 ± 3.1 (27.0–33.1)	1.4 ± 0.9 (0.5–2.3)	31 ± 3.1 (28.0–34.1)	1.6 ± 0.5 (1.1–2.1)	32 ± 1.5 (30.0–33.5)	0.8 ± 0.5 (0.3–1.3)
	72	30 ± 2.6 (28.0–32.6)	0.8 ± 0.5 (0.3–1.3)	28 ± 1.1 (27.0–29.1)	1 ± 0.2 (0.8–1.2)	30 ± 2.1 (27.8–32.1)	1 ± 0.2 (0.8–1.2)	30 ± 1.1 (29.0–31.1)	1.0 ± 0.2 (0.8–1.2)	28 ± 4.1 (24.0–32.1)	0.8 ± 0.2 (0.6–1.0)
Middle (1000 mg/kg b.w./day)	48	35 ± 1.2 (34.0–36.2)	1.4 ± 0.3 (1.1–1.7)	32 ± 2.1 (30.0–34.1)	1.6 ± 0.9 (0.9–2.5)	34 ± 2.1 (32.0–36.1)	1.4 ± 0.8 (0.6–2.2)	30 ± 4.1 (26.0–34.1)	1.2 ± 0.5 (0.7–1.7)	33 ± 2.8 (30.0–35.8)	1.8 ± 0.6 (1.2–2.4)
	72	31 ± 3.1 (28.0–30.0)	1.4 ± 0.3 (1.1–1.7)	35 ± 1.0 (34.0–36.0)	1.4 ± 0.3 (1.1–1.7)	29 ± 2.0 (27.0–31.0)	1.6 ± 0.3 (1.3–2.0)	32 ± 2.0 (30.0–34.0)	1.0 ± 0.3 (0.7–1.3)	35 ± 1.2 (33.5–36.2)	1.0 ± 0.3 (0.7–1.3)
High (2000 mg/kg b.w./day)	48	30 ± 3.4 (27.4–34.0)	0.8 ± 0.5 (0.3–1.3)	33 ± 0.5 (32.0–34.0)	0.4 ± 0.5 (0.0–0.9)	30 ± 0.5 (29.0–31.0)	1.2 ± 0.5 (0.7–1.7)	30 ± 0.5 (29.0–31.0)	0.4 ± 0.5 (0.0–1.0)	31 ± 2.1 (29.0–33.1)	1.0 ± 0.5 (0.5–1.5)
	72	30 ± 0.5 (29.0–31.0)	1.4 ± 0.3 (1.1–1.7)	30 ± 3.4 (26.0–33.4)	1.4 ± 0.3 (1.1–1.7)	31 ± 2.4 (28.5–33.4)	1.2 ± 0.2 (1.0–1.4)	29 ± 2.4 (26.5–31.4)	1.4 ± 0.3 (1.1–1.7)	30 ± 3.4 (26.5–33.4)	0.8±0.3 (0.5–1.1)
Negative control ^a^	48	33 ± 2.8 (30.0–36.0)	0.8 ± 0.5 (0.3–1.3)	35 ± 4.5 (30.0–39.5)	0.4±0.5 (0.0–0.9)	34 ± 4.5 (30.0–38.5)	0.6±0.6 (0.0–1.2)	33 ± 2.5 (31.0–35.6)	0.6 ± 0.5 (0.1–1.1)	35±4.4 (30.0–39.5)	1.4 ± 0.5 (0.9–2.0)
	72	32 ± 1.5 (30.0–33.5)	1.2 ± 0.3 (0.9–1.5)	30 ± 2.5 (27.5–32.5)	1.2 ± 0.3 (0.9–1.5)	31 ± 2.5 (28.5–33.5)	1.4 ± 0.5 (0.9–2.0)	31 ± 1.5 (29.0–32.5)	1.4 ± 0.5 (0.9–2.0)	30 ± 2.6 (27.0–33.0)	1.2 ± 0.3 (0.9–1.5)
Positive control ^b^	48	18 ± 1.2 (16.5–19.2)	20 ± 2.3 (18.0–22.3)	16 ± 1.2* (14.5–17.2)	18±2.3 (15.7–20.3)	18 ± 1.2 (16.5–19.2)	19 ± 2.3 (16.7–21.3)	15 ± 1.2 (14.0–16.2)	18 ± 3.2 (15.0–21.2)	17 ± 1.2 (16.0–18.2)	18 ± 2.3* (15.7–20.3)
	72	10 ± 2.1 (8.1–12.1)	‐	9 ± 4.1 (5.0–13.1)	‐	10 ± 4.1 (6.0–14.1)	‐	9 ± 4.1 (5.0–13.1)	‐	9 ± 4.1 (5.0–13.1)	‐

Note

Data were presented as the mean ±SD (*n* = 5).

Abbreviations: MN, micronucleus; RBC, total erythrocyte; RET, reticulocyte.

*A significant difference compared to the negative control (*p *< 0.05).

^a^ Sterile water was used as a negative control.

^b^ 2 μM mitomycin C induction

### 3.4 Oral toxicity assay

During the study period, after 28 days of repeated oral administration of SCPs, no clinical signs of abnormality, ophthalmological abnormalities, or mortality were detected in the treatment groups (500, 1000, and 2000 mg/kg body weight). Though bodyweight gradually increased during the study period in the treatment groups; however, this amount was not significant (data not shown). Water and food intake in the groups treated with three doses of SCPs were not significantly different from the control group (Table 8). Statistical analysis was performed and did not observe a significant difference in the mean between male or female mice fed with control or three doses of SCPs mixtures.

**TABLE 8:** The specific growth rate (SGR) (mean ±S.D) of mice fed with a mixture of SCPs at different doses for 28 days

Dose (mg/kg b.w./day)	Week 1		Week 2		Week 3		Week 4	
	Male	Female	Male	Female	Male	Female	Male	Female
Low dose (500)	30.67 ± 3.25	25.66 ± 1.25	32.3 ± 2.3	35.30 ± 1.2	35.6 ± 3.3	29.33 ± 8.6	35.6 ± 6.6	33.2 ± 1.2
Middle dose (1000)	25.37 ± 9.26	30.02 ± 8.66	30.3 ± 3.3	31.33 ± 2.3	33.31 ± 3.6	37 ± 3.9	32.6 ± 2.1	35.9 ± 1.0
High dose (2000)	28.67 ± 1.26	28.67 ± 9.0	31.0 ± 6.36	29.9.2 ± 6.3	36.23 ± 2.3	35.9 ± 3.9	35.3 ± 1.2	34.8 ± 1.5
Control	30.58 ± 1.36	29.0 ± 2.36	31 ± 2.0	32.56 ± 2.3	35.95 ± 3.6	38 ± 2.3	36.3 ± 1.2	36.5 ± 2.9

## 4 DISCUSSION

Over the past few decades, the use of probiotic microorganisms in various foods, especially dairy products, have significantly increased. *Lactobacillus* and Bifidobacterium are probiotic bacteria that both make up the natural flora of the human gut and have many beneficial effects on human health.^23^ In recent years, new probiotic species, especially native probiotic species, have become commercially available as effective supplements and foods used in industry and medicine. Therefore, the efficacy and safety of new probiotics must be appropriately tested.^24^

The primary assay revealed no pronounced toxicity in experimental strains at concentrations up to 5 mg/plate, this concentration was set as the highest dose, and the Ames trial was performed with its serial dilutions. One of the essential non‐clinical safety examinations is the Ames test, which detects mutagenic effects of food, chemicals, and plant extracts with high predictive results.^25^ This test could be performed using different *S*. *typhimurium* and *E*. *coli* testers with and without S9 metabolic activation. Ames test could detect indirect mutants in the presence of S9 metabolic activation; however, in the absence of S9 metabolic activation, it could only detect direct mutants.^26^ Studies on these mutations have shown that in cases where the mutagen is directly involved, it interacts directly with DNA and their reactive metabolites.^27^

Here, *S*. *typhimurium* TA98 and TA100 strains were used with various mutations (*hisD3052*, *hisG46*, *hisC3076*, and *his428*) in the histidine operon genes because of the availability of strains in similar studies by Park et al. and Shon et al..^28^, ^29^ *S*. *typhimurium* strains TA98 and TA100 have a deletion mutation in the *uvrB* gene to hold adducts generated with examination chemicals, such as *gal*, *chl*, and, *bio* genes.^9^, ^30^ Other LAB strains, such as multispecies probiotics, contain *L*. *rhamnosus* LCR177, *B*. *adolescentis* BA286, and *Pediococcus acidilactici* PA318,^31^ *Lactobacillus mali* APS1^32^ on worked with *S*. *typhimurium* strains TA97a, TA98, TA100, TA102, and TA1535 and also other LAB *B*. *mojavensis* KJS‐3 in TA98, TA100, TA1535, and TA1537,^13^ so showed no mutagenic activity under analogous conditions based on the OECD Guidelines.

According to OECD guidelines for chromosomal aberration test, five concentrations (5.0, 2.5, 1.25, 0.625, and 0.3125 mg/ml) of lactobacilli strains with or without S9 were used for cytotoxicity evaluation and incubated with CHO‐K1 cell for a short (6 h) and long (24 h) period of time, exhibiting no mutagenic activity under similar conditions. This had a short population double period and a permanent karyotype of chromosome 20/21. Karyotype constancy safeguards the functional monosonic situation of the Hypoxanthine‐guanine phosphoribosyl transferase (HGPRT) gene.^33^

Besides, metabolic activation of the test substances did not intrude the mitotic operation or cell cycle progress. In brief, this information shows that the SCPs did not induce CA in cultured mammalian somatic cells under the experimental conditions. Studies have shown that the use of *Lactobacillus* spp, either live or heat‐killed, at a dose of 0.3125 to 5 mg/mL does not lead to a significant increase in CA frequency in mammalian cells with CA compared to positive control.^32^, ^34^, ^35^ In contrast, in another study, *L*. *acidophilus* strain demonstrated a low level of anti‐mutagenic activity.^30^ Although the results showed no mutagenicity for the strains tested, the CA test is still not sufficient to challenge the validity of these samples’ conventional reverse mutation test. Other in vivo tests, such as micronucleus assays in mice, are needed to evaluate the genetic toxicity and clastogenicity of the strains more accurately.

The study of micronucleus counting is an indirect method for investigating the possible genotoxic damage at the chromosomal level.^36^ The results of our study showed that there were no genotoxic reactions in empirical animals at various maximum doses, which is consistent with the findings of various equivalent in vivo genotoxic studies on probiotics.^32^, ^34^, ^35^

Furthermore, consistent with other studies,^32^, ^36^, ^37^, ^38^28‐day administration of the tested *Lactobacillus* strains in three doses does not cause any oral toxicity or mortality in animals.

## 5 CONCLUSION

Using OECD guidelines protocols (No. 471,473, 474) for testing chemicals, this study showed that native *Lactobacillus* strains isolated from feces had no mutagenic or genotoxic effects at all doses tested using bacterial reverse mutation, chromosome aberration, micronucleus in mice, and oral toxicity assays. In this study, we assessed the Ames test using *S*. *typhimurium* strains TA98, TA100, and to confirm the results in the future, three more *S*. *typhimurium* strains should be examined. Investigating the mechanism of anti‐mutagenic properties may have important implications for future research on probiotic bacteria and their ability to reduce the risk of cancer. This study showed that these SCPs are safe in the range of concentrations evaluated in these experiments. Nevertheless, more future in‐depth studies on 90‐day oral toxicity or other concentrations of SCPs should be performed.

## AUTHOR CONTRIBUTIONS

AD and MT initiated the idea of this study. SM and RGZ developed the first and final draft of the manuscript. ME developed the second draft of the manuscript. All figures and tables were designed and checked by AD, MT, and MK. AD wrote the manuscript and carried out the data analysis. MT and MK critically reviewed and revised the manuscript. All authors reviewed and contributed to the revisions and finalized the drafts.

## CONFLICT OF INTEREST

The authors have no conflict of interest to declare.

## Supporting information

Fig S1

Table S1‐S4

## Data Availability

The data that support the findings of this study are available from the corresponding author upon reasonable request.
