# Supplementary figures and images for: Safety evaluation of mutagenicity, genotoxicity, and cytotoxicity of Lactobacillus spp. isolates as probiotic candidates
Source: J Clin Lab Anal. 2022 May 17;36(7):e24481. doi: 10.1002/jcla.24481 (PMC9279957; doi:10.1002/jcla.24481)

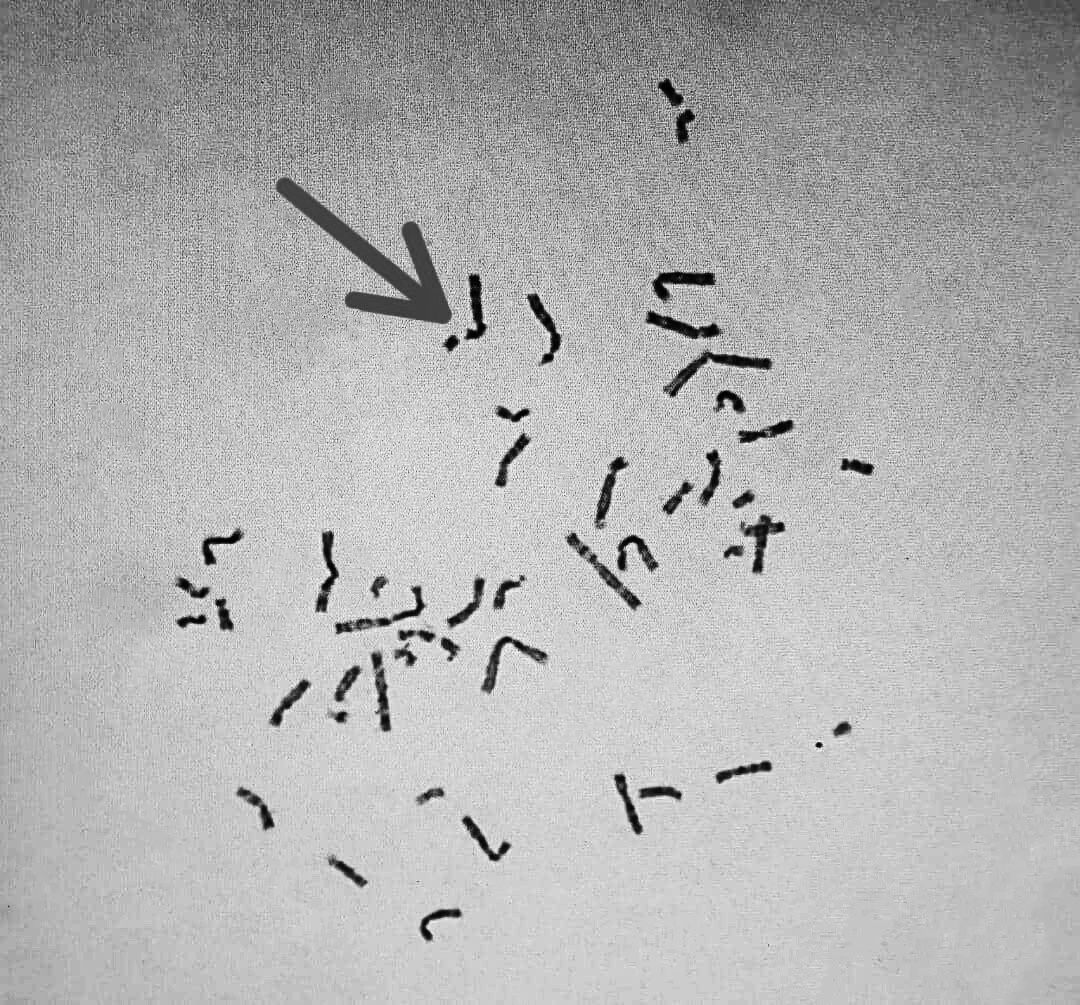

Supplement: Supplementary file 1 — Fig S1 [file JCLA-36-e24481-s002.tiff]
