# Supplementary material for: Safety evaluation of mutagenicity, genotoxicity, and cytotoxicity of Lactobacillus spp. isolates as probiotic candidates
Source: J Clin Lab Anal. 2022 May 17;36(7):e24481. doi: 10.1002/jcla.24481 (PMC9279957; doi:10.1002/jcla.24481)
Supplement: Supplementary file 2 — Table S1‐S4 [file JCLA-36-e24481-s001.docx]

***Chromosome Aberration Test***

**Table S1-A**

Number of structural and numerical chromosome aberrations induced in CHO-K1 cells by exposure to *L. plantrum 42* for 6 h period in the absence of metabolic activation.

| ***L. plantrum 42* (mg/mL (** | **Number of cells with structural chromosome aberrations (% in brackets)** | | | | | | | **Number of cells with**  **numerical aberrations (%)** | | **Survival**  **ratio (%)** |
| --- | --- | --- | --- | --- | --- | --- | --- | --- | --- | --- |
|  | **Observed** | **Chromatid**  **breaks** | **Chromatid**  **exchanges** | **Chromosome**  **breaks** | **Chromosome**  **exchanges** | **Gaps or**  **Others** | **Total** | **Polyploids** | **Endoreduplicated**  **cells** |  |
| **Negative control** | 200 | 0(0.0) | 0(0.0) | 2(1.0) | 0(0.0) | 0(0.0) | 2(1.0) | 2(1.0) | 0(0.0) | 100 |
| **0.3125** | 200 | 0(0.0) | 0(0.0) | 0(0.0) | 0(0.0) | 0(0.0) | 0(0.0) | 1(0.5) | 0(0.0) | 98 |
| **0.625** | 200 | 0(0.0) | 0(0.0) | 0(0.0) | 0(0.0) | 0(0.0) | 0(0.0) | 0(0.0) | 0(0.0) | 82 |
| **1.25** | 200 | 0(0.0) | 0(0.0) | 0(0.0) | 0(0.0) | 1(0.5) | 1(0.5) | 0(0.0) | 0(0.0) | 71 |
| **2.5** | 200 | 0(0.0) | 2(1.0) | 0(0.0) | 0(0.0) | 0(0.0) | 2(1.0) | 1(0.5) | 0(0.0) | 85 |
| **5.0** | 200 | 0(0.0) | 0(0.0) | 0(0.0) | 0(0.0) | 0(0.0) | 0(0.0) | 0(0.0) | 0(0.0) | 70 |
| **Positive control (MMC^a^ at 0.1** μg **/mL)** | 200 | 85(42.5) | 33(16.5) | 0(0.0) | 20(10.0) | 0(0.0) | 138(69) | 1(0.5) | 0(0.0) | 66 |

**^a^ MMC, mitomycin C.**

**Table S1-B**

Number of structural and numerical chromosome aberrations induced in CHO-K1 cells by exposure to *L. plantrum* ***42*** for 6 h period in the presence of metabolic activation.

| ***L. plantrum 42* (mg/mL(** | **Number of cells with structural chromosome aberrations (% in brackets)** | | | | | | | **Number of cells with**  **numerical aberrations (%)** | | **Survival**  **ratio (%)** |
| --- | --- | --- | --- | --- | --- | --- | --- | --- | --- | --- |
|  | **Observed** | **Chromatid**  **breaks** | **Chromatid**  **exchanges** | **Chromosome**  **breaks** | **Chromosome**  **exchanges** | **Gaps or**  **Others** | **Total** | **Polyploids** | **Endoreduplicated**  **Cells** |  |
| **Negative control** | 200 | 0(0.0) | 0(0.0) | 1(0.5) | 1(0.5) | 0(0.0) | 2(1.0) | 1(0.5) | 0(0.0) | 100 |
| **0.3125** | 200 | 0(0.0) | 0(0.0) | 1(0.5) | 0(0.0) | 0(0.0) | 1(0.5) | 1(0.5) | 0(0.0) | 95 |
| **0.625** | 200 | 0(0.0) | 0(0.0) | 0(0.0) | 0(0.0) | 0(0.0) | 0(0.0) | 0(0.0) | 0(0.0) | 85 |
| **1.25** | 200 | 0(0.0) | 0(0.0) | 0(0.0) | 0(0.0) | 2(1.0) | 2(1.0) | 3(1.5) | 0(0.0) | 92 |
| **2.5** | 200 | 0(0.0) | 0(0.0) | 0(0.0) | 0(0.0) | 1(0.5) | 1(0.5) | 0(0.0) | 0(0.0) | 77 |
| **5.0** | 200 | 0(0.0) | 1(0.5) | 0(0.0) | 0(0.0) | 0(0.0) | 1(0.5) | 0(0.0) | 0(0.0) | 89 |
| **Positive control (**CPP **^a^ at** 80 𝜇M**)** | 200 | 0(0.0) | 0(0.0) | 74(37) | 54(27) | 0(0.0) | 128(64) | 1(0.5) | 0(0.0) | 73 |

**^a^CPP,** cyclophosphamide monohydrate

**Table S1-C**

Number of structural and numerical chromosome aberrations induced in CHO-K1 cells by exposure to *L. plantrum 42* for 24 h in the absence of metabolic activation.

| ***L. plantrum 42* (mg/mL(** | **Number of cells with structural chromosome aberrations (% in brackets)** | | | | | | | **Number of cells with**  **numerical aberrations (%)** | | **Survival**  **ratio (%)** |
| --- | --- | --- | --- | --- | --- | --- | --- | --- | --- | --- |
|  | **Observed** | **Chromatid**  **breaks** | **Chromatid**  **exchanges** | **Chromosome**  **breaks** | **Chromosome**  **exchanges** | **Gaps or**  **Others** | **Total** | **Polyploids** | **Endoreduplicated**  **cells** |  |
| **Negative control** | 200 | 0(0.0) | 0(0.0) | 0(0.0) | 0(0.0) | 0(0.0) | 0(0.0) | 0(0.0) | 0(0.0) | 100 |
| **0.3125** | 200 | 0(0.0) | 0(0.0) | 0(0.0) | 0(0.0) | 0(0.0) | 0(0.0) | 1(0.5) | 0(0.0) | 93 |
| **0.625** | 200 | 0(0.0) | 0(0.0) | 0(0.0) | 0(0.0) | 0(0.0) | 0(0.0) | 3(1.5) | 0(0.0) | 83 |
| **1.25** | 200 | 0(0.0) | 0(0.0) | 0(0.0) | 1(0.5) | 1(0.5) | 2(1.0) | 0(0.0) | 0(0.0) | 78 |
| **2.5** | 200 | 0(0.0) | 0(0.0) | 0(0.0) | 0(0.0) | 2(1.0) | 2(1.0) | 1(0.5) | 0(0.0) | 68 |
| **5.0** | 200 | 0(0.0) | 0(0.0) | 1(0.5) | 0(0.0) | 0(0.0) | 1(0.5) | 0(0.0) | 0(0.0) | 70 |
| **Positive control (MMC^a^ at 0.05** μg **/mL)** | 200 | 0(0.0) | 0(0.0) | 38(19.0) | 38(19.0) | 64(32) | 140(70.0) | 2(1.0) | 0(0.0) | 65 |

**^a^ MMC, mitomycin C.**

**Table S2-A**

Number of structural and numerical chromosome aberrations induced in CHO-K1 cells by exposure to *L. rhamnosu 195* for 6 h period in the absence of metabolic activation

| ***L. rhamnosu 195* (mg/mL (** | **Number of cells with structural chromosome aberrations (% in brackets)** | | | | | | | **Number of cells with**  **numerical aberrations (%)** | | **Survival**  **ratio (%)** |
| --- | --- | --- | --- | --- | --- | --- | --- | --- | --- | --- |
|  | **Observed** | **Chromatid**  **breaks** | **Chromatid**  **exchanges** | **Chromosome**  **breaks** | **Chromosome**  **exchanges** | **Gaps or**  **Others** | **Total** | **Polyploids** | **Endoreduplicated**  **cells** |  |
| **Negative control** | 200 | 0(0.0) | 2(1.0) | 0(0.0) | 0(0.0) | 0(0.0) | 2(1.0) | 1(0.5) | 0(0.0) | 100 |
| **0.3125** | 200 | 0(0.0) | 0(0.0) | 1(0.5) | 0(0.0) | 0(0.0) | 1(0.5) | 0(0.0) | 0(0.0) | 88 |
| **0.625** | 200 | 0(0.0) | 0(0.0) | 0(0.0) | 0(0.0) | 0(0.0) | 0(0.0) | 0(0.0) | 0(0.0) | 82 |
| **1.25** | 200 | 0(0.0) | 0(0.0) | 0(0.0) | 0(0.0) | 1(0.5) | 1(0.5) | 1(0.5) | 0(0.0) | 86 |
| **2.5** | 200 | 0(0.0) | 0(0.0) | 0(0.0) | 0(0.0) | 0(0.0) | 0(0.0) | 1(0.5) | 0(0.0) | 85 |
| **5.0** | 200 | 0(0.0) | 0(0.0) | 0(0.0) | 0(0.0) | 0(0.0) | 0(0.0) | 0(0.0) | 0(0.0) | 78 |
| **Positive control (MMC^a^ at 0.1** μg **/mL)** | 200 | 95(47.5) | 33(16.5) | 2(1.0) | 2 (1.0) | 2(1.0) | 134(67) | 1(0.5) | 2(1.0) | 69 |

**^a^MMC, mitomycin C.**

**Table S2-B**

Number of structural and numerical chromosome aberrations induced in CHO-K1 cells by exposure to *L. rhamnosu 195* for 6 h period in the presence of metabolic activation.

| ***L. rhamnosu 195* (mg/mL(** | **Number of cells with structural chromosome aberrations (% in brackets)** | | | | | | | **Number of cells with**  **numerical aberrations (%)** | | **Survival**  **ratio (%)** |
| --- | --- | --- | --- | --- | --- | --- | --- | --- | --- | --- |
|  | **Observed** | **Chromatid**  **breaks** | **Chromatid**  **exchanges** | **Chromosome**  **breaks** | **Chromosome**  **exchanges** | **Gaps or**  **Others** | **Total** | **Polyploids** | **Endoreduplicated**  **cells** |  |
| **Negative control** | 200 | 0(0.0) | 0(0.0) | 0(0.0) | 0(0.0) | 0(0.0) | 0(0.0) | 0(0.0) | 0(0.0) | 100 |
| **0.3125** | 200 | 0(0.0) | 0(0.0) | 1(0.5) | 0(0.0) | 0(0.0) | 1(0.5) | 1(0.5) | 0(0.0) | 96 |
| **0.625** | 200 | 0(0.0) | 0(0.0) | 0(0.0) | 0(0.0) | 0(0.0) | 0(0.0) | 0(0.0) | 0(0.0) | 85 |
| **1.25** | 200 | 0(0.0) | 0(0.0) | 0(0.0) | 0(0.0) | 0(0.0) | 0(0.0) | 2(1.0) | 0(0.0) | 88 |
| **2.5** | 200 | 0(0.0) | 0(0.0) | 0(0.0) | 0(0.0) | 1(0.5) | 1(0.5) | 1(0.5) | 0(0.0) | 87 |
| **5.0** | 200 | 0(0.0) | 0(0.0) | 0(0.0) | 0(0.0) | 0(0.0) | 0(0.0) | 0(0.0) | 0(0.0) | 79 |
| **Positive control (**CPP **^a^ at** 80 𝜇M**)** | 200 | 0(0.0) | 2(1.0) | 84(42) | 0(0.0) | 0(0.0) | 86(43) | 3(1.5) | 2(1.0) | 61 |

**^a^CPP,** cyclophosphamide monohydrate

**Table S2-C**

Number of structural and numerical chromosome aberrations induced in CHO-K1 cells by exposure to *L. rhamnosu 195* for 24 h in the absence of metabolic activation.

| ***L. rhamnosu 195* (mg/mL(** | **Number of cells with structural chromosome aberrations (% in brackets)** | | | | | | | **Number of cells with**  **numerical aberrations (%)** | | **Survival**  **ratio (%)** |
| --- | --- | --- | --- | --- | --- | --- | --- | --- | --- | --- |
|  | **Observed** | **Chromatid**  **breaks** | **Chromatid**  **exchanges** | **Chromosome**  **breaks** | **Chromosome**  **exchanges** | **Gaps or**  **Others** | **Total** | **Polyploids** | **Endoreduplicated**  **cells** |  |
| **Negative control** | 200 | 0(0.0) | 0(0.0) | 2(1.0) | 0(0.0) | 0(0.0) | 2(1.0) | 0(0.0) | 0(0.0) | 100 |
| **0.3125** | 200 | 0(0.0) | 0(0.0) | 0(0.0) | 0(0.0) | 0(0.0) | 0(0.0) | 1(0.5) | 0(0.0) | 87 |
| **0.625** | 200 | 0(0.0) | 0(0.0) | 0(0.0) | 0(0.0) | 0(0.0) | 0(0.0) | 2(1.0) | 0(0.0) | 80 |
| **1.25** | 200 | 0(0.0) | 0(0.0) | 0(0.0) | 1(0.5) | 1(0.5) | 2(1.0) | 0(0.0) | 0(0.0) | 85 |
| **2.5** | 200 | 0(0.0) | 0(0.0) | 0(0.0) | 0(0.0) | 2(1.0) | 2(1.0) | 0(0.0) | 0(0.0) | 68 |
| **5.0** | 200 | 0(0.0) | 0(0.0) | 1(0.5) | 1(0.5) | 0(0.0) | 2(1.0) | 0(0.0) | 0(0.0) | 85 |
| **Positive control (MMC^a^ at 0.05** μg **/mL)** | 200 | 93(46.5) | 0(0.0) | 25(12.5) | 25(12.5) | 0(0.0) | 143(71.5) | 2(1.0) | 1(0.5) | 75 |

**^a^ MMC, mitomycin C.**

**Table S3-A**

Number of structural and numerical chromosome aberrations induced in CHO-K1 cells by exposure to *L. brevis 205* for 6 h period in the absence of metabolic activation.

| ***L. brevis 205* (mg/mL (** | **Number of cells with structural chromosome aberrations (% in brackets)** | | | | | | | **Number of cells with**  **numerical aberrations (%)** | | **Survival**  **ratio (%)** |
| --- | --- | --- | --- | --- | --- | --- | --- | --- | --- | --- |
|  | **Observed** | **Chromatid**  **breaks** | **Chromatid**  **exchanges** | **Chromosome**  **breaks** | **Chromosome**  **exchanges** | **Gaps or**  **others** | **Total** | **Polyploids** | **Endoreduplicated**  **Cells** |  |
| **Negative control** | 200 | 0(0.0) | 0(0.0) | 1(0.5) | 0(0.0) | 0(0.0) | 1(0.5) | 0(0.0) | 0(0.0) | 100 |
| **0.3125** | 200 | 0(0.0) | 0(0.0) | 2(1.0) | 0(0.0) | 0(0.0) | 2(1.0) | 1(0.5) | 0(0.0) | 88 |
| **0.625** | 200 | 0(0.0) | 0(0.0) | 0(0.0) | 0(0.0) | 0(0.0) | 0(0.0) | 0(0.0) | 0(0.0) | 75 |
| **1.25** | 200 | 0(0.0) | 0(0.0) | 0(0.0) | 0(0.0) | 1(0.5) | 1(0.5) | 2(1.0) | 0(0.0) | 71 |
| **2.5** | 200 | 0(0.0) | 0(0.0) | 0(0.0) | 0(0.0) | 0(0.0) | 0(0.0) | 3(1.5) | 0(0.0) | 85 |
| **5.0** | 200 | 0(0.0) | 0(0.0) | 0(0.0) | 0(0.0) | 0(0.0) | 0(0.0) | 0(0.0) | 0(0.0) | 88 |
| **Positive control (MMC^a^ at 0.1** μg **/mL)** | 200 | 96(48.0) | 33(16.5) | 0(0.0) | 2(1.0) | 0(0.0) | 131(65.5) | 1(0.5) | 1(0.5) | 68 |

**^a^ MMC, mitomycin C.**

**Table S3-B**

Number of structural and numerical chromosome aberrations induced in CHO-K1 cells by exposure to *L. brevis 205* for 6 h period in the presence of metabolic activation.

| ***L. brevis 205* (mg/mL(** | **Number of cells with structural chromosome aberrations (% in brackets)** | | | | | | | **Number of cells with**  **numerical aberrations (%)** | | **Survival**  **ratio (%)** |
| --- | --- | --- | --- | --- | --- | --- | --- | --- | --- | --- |
|  | **Observed** | **Chromatid**  **breaks** | **Chromatid**  **exchanges** | **Chromosome**  **Breaks** | **Chromosome**  **exchanges** | **Gaps or**  **others** | **Total** | **Polyploids** | **Endoreduplicated**  **Cells** |  |
| **Negative control** | 200 | 0(0.0) | 0(0.0) | 1(0.5) | 1(0.5) | 0(0.0) | 2(1.0) | 1(0.5) | 0(0.0) | 100 |
| **0.3125** | 200 | 0(0.0) | 1(0.5) | 1(0.5) | 0(0.0) | 0(0.0) | 2(1.0) | 0(0.0) | 0(0.0) | 95 |
| **0.625** | 200 | 0(0.0) | 0(0.0) | 0(0.0) | 0(0.0) | 0(0.0) | 0(0.0) | 0(0.0) | 0(0.0) | 91 |
| **1.25** | 200 | 0(0.0) | 0(0.0) | 0(0.0) | 0(0.0) | 2(1.0) | 2(1.0) | 3(1.5) | 0(0.0) | 82 |
| **2.5** | 200 | 0(0.0) | 0(0.0) | 0(0.0) | 0(0.0) | 1(0.5) | 1(0.5) | 0(0.0) | 0(0.0) | 85 |
| **5.0** | 200 | 2(1.0) | 0(0.0) | 0(0.0) | 0(0.0) | 0(0.0) | 2(1.0) | 0(0.0) | 0(0.0) | 89 |
| **Positive control (**CPP **^a^ at 0.1** μg **/mL)** | 200 | 55(27.5) | 65(32.5) | 0(0.0) | 20(10.0) | 0(0.0) | 140(70.0) | 1(0.5) | 2(1.0) | 71 |

**^a^ CPP,** cyclophosphamide monohydrate

**Table S3-C**

Number of structural and numerical chromosome aberrations induced in CHO-K1 cells by exposure to *L. brevis 205* for 24 h in the absence of metabolic activation.

| ***L. brevis 205* (mg/mL(** | **Number of cells with structural chromosome aberrations (% in brackets)** | | | | | | | **Number of cells with**  **numerical aberrations (%)** | | **Survival**  **ratio (%)** |
| --- | --- | --- | --- | --- | --- | --- | --- | --- | --- | --- |
|  | **Observed** | **Chromatid**  **breaks** | **Chromatid**  **exchanges** | **Chromosome**  **Breaks** | **Chromosome**  **exchanges** | **Gaps or**  **others** | **Total** | **Polyploids** | **Endoreduplicated**  **Cells** |  |
| **Negative control** | 200 | 2(1.0) | 0(0.0) | 0(0.0) | 0(0.0) | 0(0.0) | 2(1.0) | 0(0.0) | 0(0.0) | 100 |
| **0.3125** | 200 | 0(0.0) | 0(0.0) | 0(0.0) | 0(0.0) | 2(1.0) | 2(1.0) | 1(0.5) | 0(0.0) | 85 |
| **0.625** | 200 | 0(0.0) | 0(0.0) | 0(0.0) | 0(0.0) | 0(0.0) | 0(0.0) | 1(0.5) | 0(0.0) | 80 |
| **1.25** | 200 | 0(0.0) | 0(0.0) | 0(0.0) | 0(0.0) | 0(0.0) | 0(0.0) | 0(0.0) | 0(0.0) | 78 |
| **2.5** | 200 | 0(0.0) | 0(0.0) | 0(0.0) | 0(0.0) | 2(1.0) | 2(1.0) | 2(1.0) | 0(0.0) | 80 |
| **5.0** | 200 | 0(0.0) | 0(0.0) | 0(0.0) | 0(0.0) | 0(0.0) | 0(0.0) | 0(0.0) | 0(0.0) | 88 |
| **Positive control (MMC^a^ at 0.05** μg **/mL)** | 200 | 0(0.0) | 0(0.0) | 96(48.0) | 46(23.0) | 0(0.0) | 142(71.0) | 0(0.0) | 0(0.0) | 73 |

**^a^ MMC, mitomycin C.**

**Table S4-A**

Number of structural and numerical chromosome aberrations induced in CHO-K1 cells by exposure to *L. plantrum 165* for 6 h period in the absence of metabolic activation.

| ***L. plantrum 165* (mg/mL (** | **Number of cells with structural chromosome aberrations (% in brackets)** | | | | | | | **Number of cells with**  **numerical aberrations (%)** | | **Survival**  **ratio (%)** |
| --- | --- | --- | --- | --- | --- | --- | --- | --- | --- | --- |
|  | **Observed** | **Chromatid**  **breaks** | **Chromatid**  **exchanges** | **Chromosome**  **breaks** | **Chromosome**  **exchanges** | **Gaps or**  **others** | **Total** | **Polyploids** | **Endoreduplicated**  **Cells** |  |
| **Negative control** | 200 | 0(0.0) | 0(0.0) | 0(0.0) | 0(0.0) | 0(0.0) | 0(0.0) | 0(0.0) | 0(0.0) | 100 |
| **0.3125** | 200 | 1(0.5) | 0(0.0) | 0(0.0) | 0(0.0) | 0(0.0) | 1(0.5) | 2(1.0) | 0(0.0) | 95 |
| **0.625** | 200 | 0(0.0) | 1(0.5) | 0(0.0) | 0(0.0) | 0(0.0) | 1(0.5) | 2(1.0) | 0(0.0) | 85 |
| **1.25** | 200 | 0(0.0) | 0(0.0) | 0(0.0) | 0(0.0) | 0(0.0) | 0(0.0) | 1(0.5) | 0(0.0) | 71 |
| **2.5** | 200 | 0(0.0) | 2(1.0) | 0(0.0) | 0(0.0) | 0(0.0) | 2(1.0) | 1(0.5) | 0(0.0) | 85 |
| **5.0** | 200 | 0(0.0) | 0(0.0) | 0(0.0) | 0(0.0) | 0(0.0) | 0(0.0) | 0(0.0) | 0(0.0) | 80 |
| **Positive control (MMC^a^at 0.1** μg **/mL)** | 200 | 2(1.0) | 20(10.0) | 0(0.0) | 20(10.0) | 96(48.0) | 138(69) | 1(0.5) | 2(1.0) | 70 |

**^a^ MMC, mitomycin C.**

**Table S4-B**

Number of structural and numerical chromosome aberrations induced in CHO-K1 cells by exposure *L. plantrum 165* for 6 h period in the presence of metabolic activation.

| ***L. plantrum 165* (mg/mL(** | **Number of cells with structural chromosome aberrations (% in brackets)** | | | | | | | **Number of cells with**  **numerical aberrations (%)** | | **Survival**  **ratio (%)** |
| --- | --- | --- | --- | --- | --- | --- | --- | --- | --- | --- |
|  | **Observed** | **Chromatid**  **breaks** | **Chromatid**  **exchanges** | **Chromosome**  **Breaks** | **Chromosome**  **exchanges** | **Gaps or**  **others** | **Total** | **Polyploids** | **Endoreduplicated**  **cells** |  |
| **Negative control** | 200 | 0(0.0) | 0(0.0) | 1(0.5) | 1(0.5) | 0(0.0) | 2(1.0) | 2(1.0) | 0(0.0) | 100 |
| **0.3125** | 200 | 0(0.0) | 0(0.0) | 1(0.5) | 0(0.0) | 0(0.0) | 1(0.5) | 1(0.5) | 0(0.0) | 95 |
| **0.625** | 200 | 0(0.0) | 0(0.0) | 0(0.0) | 0(0.0) | 0(0.0) | 0(0.0) | 0(0.0) | 0(0.0) | 87 |
| **1.25** | 200 | 0(0.0) | 0(0.0) | 0(0.0) | 0(0.0) | 2(1.0) | 2(1.0) | 0(0.0) | 0(0.0) | 87 |
| **2.5** | 200 | 0(0.0) | 0(0.0) | 0(0.0) | 0(0.0) | 1(0.5) | 1(0.5) | 0(0.0) | 0(0.0) | 77 |
| **5.0** | 200 | 0(0.0) | 0(0.0) | 0(0.0) | 0(0.0) | 0(0.0) | 0(0.0) | 0(0.0) | 0(0.0) | 85 |
| **Positive control (**CPP **^a^ at 0.1** μg **/mL)** | 200 | 0(0.0) | 0(0.0) | 74(37) | 54(27) | 0(0.0) | 128(64) | 1(0.5) | 0(0.0) | 68 |

**^a^ CPP,** cyclophosphamide monohydrate

Table S4-C

Number of structural and numerical chromosome aberrations induced in CHO-K1 cells by exposure to *L. plantrum 165* for 24 h in the absence of metabolic activation.

| ***L. plantrum 165* (mg/mL(** | **Number of cells with structural chromosome aberrations (% in brackets)** | | | | | | | **Number of cells with**  **numerical aberrations (%)** | | **Survival**  **ratio (%)** |
| --- | --- | --- | --- | --- | --- | --- | --- | --- | --- | --- |
|  | **Observed** | **Chromatid**  **breaks** | **Chromatid**  **exchanges** | **Chromosome**  **Breaks** | **Chromosome**  **exchanges** | **Gaps or**  **others** | **Total** | **Polyploids** | **Endoreduplicated**  **cells** |  |
| **Negative control** | 200 | 2(1.0) | 0(0.0) | 0(0.0) | 0(0.0) | 0(0.0) | 2(1.0) | 0(0.0) | 0(0.0) | 100 |
| **0.3125** | 200 | 2(1.0) | 0(0.0) | 0(0.0) | 0(0.0) | 0(0.0) | 2(1.0) | 0(0.0) | 0(0.0) | 89 |
| **0.625** | 200 | 0(0.0) | 0(0.0) | 0(0.0) | 0(0.0) | 0(0.0) | 0(0.0) | 0(0.0) | 0(0.0) | 78 |
| **1.25** | 200 | 0(0.0) | 0(0.0) | 0(0.0) | 1(0.5) | 1(0.5) | 2(1.0) | 0(0.0) | 0(0.0) | 85 |
| **2.5** | 200 | 0(0.0) | 0(0.0) | 0(0.0) | 0(0.0) | 2(1.0) | 2(1.0) | 3(1.5) | 0(0.0) | 80 |
| **5.0** | 200 | 0(0.0) | 0(0.0) | 1(0.5) | 0(0.0) | 0(0.0) | 1(0.5) | 0(0.0) | 0(0.0) | 80 |
| **Positive control (MMC^a^ at 0.05** μg **/mL)** | 200 | 0(0.0) | 36(18.0) | 38(19.0) | 0(0.0) | 0(0.0) | 74(37.0) | 0(0.0) | 1(0.5) | 68 |

**^a^ MMC, mitomycin C.**
